# Herbal medicines for treating tic disorders: a systematic review of randomised controlled trials

**DOI:** 10.1186/1749-8546-9-6

**Published:** 2014-02-07

**Authors:** Yun Hee Kim, Chang-Gue Son, Bon-Cho Ku, Hye Won Lee, Hyun Sook Lim, Myeong Soo Lee

**Affiliations:** 1 Korean Institute of Oriental Medicine, Daejeon, South Korea; 2 Liver & Immunology Research Center, Daejeon Oriental Hospital of Daejeon University, Daejeon, South Korea; 3 Department of Nursing, Howon University, Kunsan, South Korea

## Abstract

**Background:**

It was reported that 64% of tic disorder patients used complementary and alternative medicine. This review aims to evaluate the efficacy of herbal medicines in treating tic disorders.

**Methods:**

We searched eight databases including MEDLINE and CINAHL from their respective inceptions up to September 2013. The search terms were related to the concept of “herbal medicine” AND “tic disorder OR Tourette’s syndrome”. We included randomised controlled trials (RCTs) of any type of herbal medicines. We assessed the methodological quality of the trials according to the Cochrane risk of bias criteria.

**Results:**

Sixty one studies were identified, and four RCTs met the inclusion criteria. Two types of herbal medicines, Qufeng Zhidong Recipe (QZR) decoction and Ningdong (ND) granules, were used in the included RCTs. All four RCTs had a high risk of bias. Two RCTs tested the effects of QZR on the Yale Global Tic Severity Scale (YGTSS) score and response rate compared with conventional medicine. The meta-analysis showed significant effects of QZR on the YGTSS score with high statistical heterogeneity (n = 142; weighted mean difference: −18.34; 95% confidence interval (CI): −23.07 to −13.60; I^2^ = 97%) and the response rate (n = 142; risk ratio: 1.69; 95% CI: 1.39 to 2.06; I^2^ = 0%). One RCT compared ND granules with placebo and showed significant effects on the YGTSS score and response rate. The other RCT show significant effects of ND granules plus conventional medicine on the response rate compared with conventional medicine only.

**Conclusion:**

This systematic review provided first piece of limited meta-analytic evidence for the effectiveness of herbal medicines in improving the symptoms of tic disorders.

## Background

Tic disorders, which are characterised by sudden, repetitive, nonrhythmic motor movement or vocalisation, are observed in approximately 6–20% of children in worldwide [1]. The tic disorder spectrum ranges from mild to more severe, and the disorders are classified by duration and severity into transient tic, chronic tic, or Tourette’s syndrome [2-4]. Although functional impairment is relatively rare, tic disorders can affect academic achievement, sleep quality, and emotional status (including anxiety or depression), and in severe cases, they can cause social withdrawal [5].

The exact cause of tic disorders is unknown. Until the 1960s, psychogenic factors were regarded as the primary cause [6]. Recently, the domain of tic disorders has moved to the border between neurology and psychology [6]. Neuroimaging evidence suggested that an imbalance in the dopaminergic system increased the expression of presynaptic dopamine transporters and causes excessive dopaminergic innervation [7].

Antipsychotic drugs that function as dopamine receptor antagonists are used to treat tic disorders [1,6], and the efficacy of antipsychotic drugs, such as haloperidol and pimozide, has been demonstrated in clinical trials [4,7]. However, many clinicians are reluctant to use these drugs because of the potential adverse effects [6]. Tic disorder patients are increasingly using complementary and alternative medicine (CAM), and one study reported that 64% of tic disorder patients received CAM [8]. Animal studies on the effect of herbal medicines in treating tic disorders [9,10].

However, there has been no comprehensive evaluation of the clinical studies on the beneficial and adverse effects of herbal medicines on tic disorders. This systematic review of randomised controlled trials (RCTs) aims to evaluate the efficacy of herbal medicines in treating tic disorders.

## Methods

### Search methods used to identify studies

The following databases were searched from their respective inceptions up to September 2013: MEDLINE; Cumulative Index to Nursing and Allied Health Literature (CINAHL); SciVerse Scopus (SCOPUS); EBSCO Academic Search; Cochrane Central Register of Controlled Trials (CENTRAL); a Chinese database (China Network Knowledge Infrastructure [CNKI]); and two Korean databases (Oriental Medicine Advanced Searching Integrated System [OASIS] and Korean Studies Information Service System [KISS]). The search strategy is listed in Table 1. No language restrictions were imposed. Dissertations and abstracts were included.

**Table 1:** Medline (PubMed) search strategy (* used as the wildcard character Truncating search terms for searching for all terms that begin with a word)

#1	tic disorder [mh]
#2	Tourette’s syndrome [mh]
#3	1 or 2
#4	Medicine, African Traditional [mh]
#5	Medicine, Arabic [mh]
#6	Medicine, Ayurvedic [mh]
#7	Medicine, Kampo [mh]
#8	Medicine, Korean Traditional [mh]
#9	Medicine, Tibetan Traditional [mh]
#10	Medicine, Mongolian Traditional [mh]
#11	Herbal Medicine [mh]
#12	Phytotherapy [mh]
#13	Drugs, Chinese Herbal [mh]
#14	Plants, Medicinal [mh]
#15	Plant Extracts [mh]
#16	Ethnobotany [mh]
#17	Ethnopharmacology [mh]
#18	Plants [mh]
#19	Herb* [tiab]
#20	Any of 4–19
#21	3 and 20

### Selection criteria regarding the types of studies

RCTs and quasi-RCTs of the use of herbal medicines for treating tic disorders and Tourette’s syndrome were included. We included both parallel-group and crossover study designs.

### Participants

We focused on the patients with clinical diagnoses of tic disorders (persistent or provisional) or Tourette’s syndrome who met the *Diagnostic and Statistical Manual of Mental Disorders* (*DSM*) III, IV, or V criteria. Studies with both children and adults were included.

### Interventions

Studies that used herbal medicines or combination therapy with conventional medicine versus placebo or other medications were included. Combinations of herbal medicines and non-medicinal therapy, such as acupuncture, and comparisons between different types of herbal medicines were excluded. We defined herbal medicines as product decoctions, concoctions, capsules, tablets, and powders that originated from botanical sources, such as whole plants or their adjuncts [11]. However, single-chemical extracts or synthetic plant-based drugs were excluded. Only interventions that used medications taken internally were included, and other CAM treatments were excluded. There were no restrictions on frequency, dose, or duration.

### Outcome measures

The primary outcomes analysed for the review were tic severity, intensity, complexity, and interference. These outcomes were measured by the Yale Global Tic Severity Scale (YGTSS), Tic Symptom Score Scale, Tourette’s Syndrome Global Scale, and a self-rating scale (Tourette’s Syndrome Symptom List). The secondary outcomes analysed for the review were adverse events, as measured by the Treatment Emergent Symptom Scale, electroencephalograms, laboratory tests (erythrocyte sedimentation rate , kidney and liver function tests), and electrocardiogram abnormalities or changes.

### Data extraction

All articles were read by two independent reviewers (YHK, CGS) who also extracted data according to the following predefined criteria: disease severity; interventions for treatment and control groups; trial duration; outcome measurements; and recipes of herbal medicines. Any disagreement between the two reviewers (YHK, CGS) was solved by discussion.

### Assessment of risk of bias in the included studies

Two reviewers (YHK, HYL) independently assessed the risk of bias in the included studies according to the criteria set forth in the Cochrane Handbook version 5.1.0, which include random sequence generation, allocation concealment, blinding of participants and personnel, blinding of outcome assessments, incomplete outcome data, selective reporting, and other sources of bias [12]. Any differences in opinion were resolved through discussion or consultation with the third reviewer (MSL).

### Data synthesis

The values (end of treatment) of the outcome measures after treatment were used to assess differences between the herbal medicine groups and control groups. We did not include any follow-up treatment values. Weighted mean differences (WMDs) were used when studies measured the outcomes on the same scale, and standard mean differences (SMDs) were used when studies measured the outcomes on different scales using random-effects model. These values were obtained using Review Manager 5.1 (Copenhagen: The Nordic Cochrane Centre, The Cochrane Collaboration, 2011). Risk ratios (RRs) and 95% confidence intervals (CIs) were assessed for the binary data. The χ^2^, τ^2^, and Higgins I^2^ were used to assess heterogeneity. *P* < 0.05 was considered statistically significant.

## Results

### Characteristics of the studies

**Figure 1:**
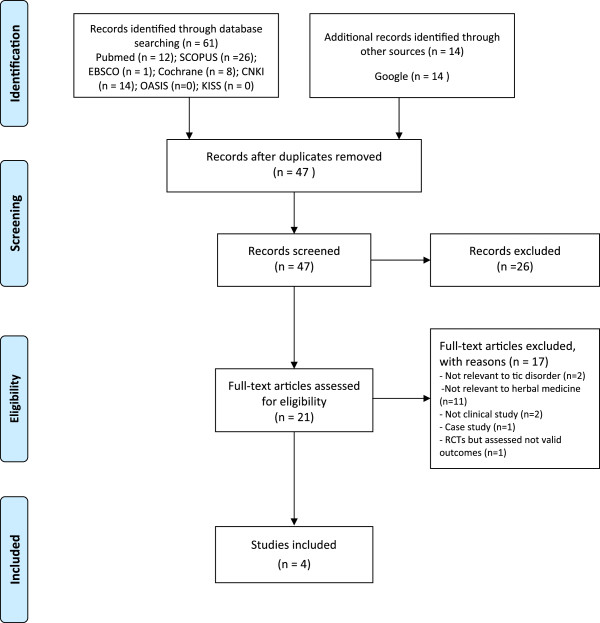
**Flow chart of the publication selection process.** RCT: randomised clinical trial.

Sixty-one papers were extracted in the initial screening of the eight databases. There were five RCT-based papers. One RCT [13] was subsequently excluded, as the assessment had no valid outcome. Four papers were ultimately selected for the review process and data analysis (Figure 1 and Table 2) [14-17]. All four studies were designed with parallel groups (only two arms), and the diagnosis was made using the *DSM-IV* criteria. One trial [16] was a randomised double-blind study, and the other three trials [14,15,17] were randomised open-label studies.

**Table 2:** Summary of RCTs using herbal medicines for tic disorders

**Author (year)**	**Sample size**	**Interventions (regimen)**	**Control (regimen)**	**Outcomes**	**Intergroup differences**	**Adverse events**
	**Conditions**					
	**Age (years)**					
	**Diagnosis criteria**					
Wu *et al.* (2009) [14]	81	(A) QZR (decoction, 200 ml daily for 24 wks, n = 40)	(B) Haloperidol and trihexyphenidyl (50 μg/kg, n = 41)	(1) YGTSS Score	(1) MD −20.73 [−21.8, –19.66], *P* < 0.05	(A) Loss of appetite
	Children with tic disorder					
	8–10					
	66/15					
	*DSM-IV* criteria					
				(2) Effective rate	(2) RR 1.72[1.32, 2.25], *P* < 0.05	(B) Weight gain and drowsiness
Wu *et al.* (2010) [15]	61	(A) QZR (decoction, 200 ml daily for 24 wks, n = 31)	(B) Haloperidol and trihexyphenidyl (50 μg/kg, n =30)	(1) YGTSS Score	(1) MD −15.9 [−17.31, −14.49], *P* < 0.05	(A) Loss of appetite
	Children with tic disorder					
	51/10					
	*DSM-IV* criteria					
				(2) Effective rate	(2) RR 1.65[1.23, 2.21], *P* < 0.05	(B) Weight gain and drowsiness
Zhao *et al.* (2010) [16]	64	(A) ND (granule capsule, 1 g daily for 8 wks, n = 33)	(B) Placebo (n = 31)	(1) YGTSS Score	(1) MD −6.52 [−9.8, –3.24], *P* < 0.001	(A) Loss of appetite, constipation
				(2) Effective rate	(2) RR4.3 [ 1.68, 11.0], *P* < 0.001	(B) None
	Children with Tourette’s syndrome					
	7–18					
	57/7					
	*DSM-IV* criteria					
Li *et al.* (2009) [17]	90	(A) ND (granules, 3–9 g daily for 24 wks, n = 60), plus (B)	(B) Haloperidol (2–6 mg, n = 30)	(1) YGTSS Score	(1) MD −4.35 [−7.34, –1.36], *P* < 0.01	(A) and (B) Drowsiness, lassitude, poor appetite
	Children with Tourette’s syndrome					
				(2) Effective rate	(2) RR1.3 [ 1.04, 1.62], *P* < 0.01	
	6–13					
	70/20					
	*DSM-IV* criteria					

In total, 296 patients participated in the four studies, and all patients were younger than 18 years. The mean sample size was 74 (range: 61–90), and the mean study duration was 20 weeks (range: 8–24 weeks). The patients in two trials [14,15] were diagnosed with tic disorders, while the other two studies [16,17] examined Tourette’s syndrome.

The four trials were conducted by two groups to evaluate two herbal decoctions (Table 3). Two studies compared *Qufeng Zhidong* Recipe (QZR) decoction with haloperidol [14,15]. In the other studies, *Ningdong* (ND) granules or ND granules plus haloperidol were compared with placebo or haloperidol alone [16,17].

**Table 3:** Preparation of herbal medicines for tic disorders

**Name of herbal drug**	**Components of the herbal medicine**	**Composition (g)**
**Type and yield**		
*Qufeng Zhidong* recipe 200 mL from a 65 g decoction	Gastrodiae Rhizoma *(Tian Ma)*	10
	Ramulus Uncariaecum Uncis *(Gou Teng)*	10
	Chaenomelis Fructus *(Mu Gua)*	10
	Lycopodii Herba (*Shen Jin Cao)*	10
	Magnoliae Flos *(Xin Yi)*	10
	Isatidis radix *(Ban Lan Gen)*	10
	Scorpio *(Quan Xie)*	5
*Ning Dong* granules 16.4 g granules from a 25 g crude herb mix	Gastrodiae Rhizoma *(Tian Ma)*	2
	Codonopsis pilosula *(Dang Shen)*	3
	*Ophiopogon japonicus (Mai Men Dong)*	2
	*Paeonia lact flora* Pall *(Bai Shao Yao)*	4
	Osdraconis *(Long Gu*)	5
	*Ostreagigas* Thunb *(Du Li)*	5
	*Pheretimaas pergillum (Di Long)*	2
	*Glycyrrhizauralensis* Fisch *(Gan Cao)*	2

### Risk of bias

As shown in Table 4, the randomisation methods were not described in two studies [15,16], while the other two trials referred to a random number table [14,17]. No details were provided for the method of allocation concealment in any of the studies. Only one trial was a double-blind study [16], and the others were open-label studies. Reporting of dropout cases was provided for three trials, but the detailed reasons were not shown [14-16], while the last trial did not report dropout cases [17]. Selective reporting bias could not be evaluated because of the unclear descriptions of the preregistered protocols.

**Table 4:** Bias risks in the included RCTs

**Study**	**Random sequence generation**	**Allocation concealment**	**Blinding of participants**	**Incomplete outcome data**	**Selective outcome reporting**	**Other bias**
Wu *et al.* (2009) [14]	L	U	H	U	U	U
Wu *et al.* (2010) [15]	U	U	H	U	U	U
Zhao *et al.* (2010) [16]	L	U	H	U	U	U
Li *et al.* (2009) [17]	U	U	L	U	U	U

*L* low risk of bias; *U* unclear risk of bias; *H* high risk of bias.

### QZR versus conventional medicine

**Figure 2:**
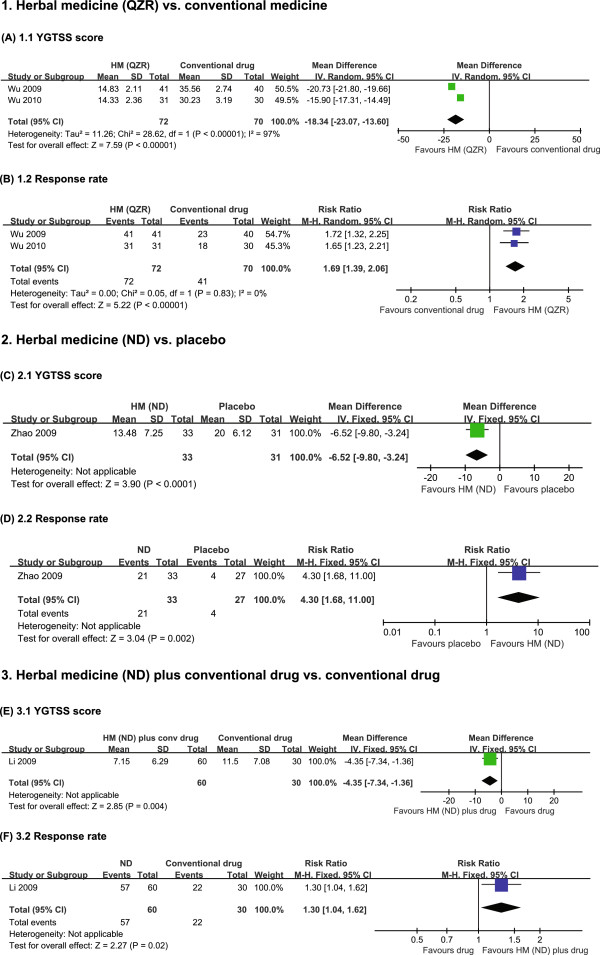
**Forest plot of the effects of herbal medicines on the Yale Global Tic Severity Scale (YGTSS) score and response rate.** HM: herbal medicine.

The two QZR trials included a single-site trial [15] and a multicentre trial [14] examining QZR versus the combination of haloperidol and trihexyphenidyl in tic disorders. In the single-site trial, the YGTSS score were decreased in both groups, but the QZR-treated group showed a significant improvement compared with the control group after 24 weeks [15]. The multicentre study found a significant improvement in the YGTSS score of the treatment group compared with the control group after administering the drugs for 24 weeks [14]. The meta-analysis showed significant superior effects of QZR on improvement of the YGTSS score compared with conventional drug treatment (haloperidol plus trihexyphenidyl) (n = 142; WMD: −18.34; 95% CI: −23.07 to −13.60; *P* < 0.00001; Figure 2A) with high heterogeneity (τ^2^ = 11.26; χ^2^ = 28.62; *P* < 0.00001; I^2^ = 97%). QZR showed a significant improvement compared with conventional medicine in response rate (n = 142; RR: 1.69; 95% CI: 1.39 to 2.06; heterogeneity: τ^2^ = 0; χ^2^ = 0.05; *P* = 0.82; I^2^ = 0%; Figure 2B).

### ND granules versus placebo

One RCT evaluated the effect of ND granules in treating Tourette’s syndrome compared with placebo [16]. Compared with the placebo group, administration of ND granules for 8 weeks significantly reduced the YGTSS score (n = 64; MD: −6.52; 95% CI: −9.80 to −3.24; *P* < 0.0001; Figure 2C) and the response rate (n = 64; RR: 4.30; 95% CI: 1.68 to 11.0; Figure 2D).

### ND granules plus haloperidol versus haloperidol only

One RCT tested ND granules plus haloperidol for treating Tourette’s syndrome compared with haloperidol only [17]. After 24 weeks of treatment, the group that received ND granules plus haloperidol showed significant decreases in the total YGTSS score (n = 90; MD: –4.35; 95% CI: −7.34 to −1.36; Figure 2E) and the response rate (n = 90; RR: 1.30; 95% CI: 1.04 to 1.62; Figure 2F) compared with the haloperidol only group.

### Adverse effects of QZR and ND granules

In the two QZR trials, no significant adverse events were observed, except for slight loss of appetite, whereas the participants in the haloperidol group experienced weight gain, hypopraxia, and drowsiness despite using trihexyphenidyl [14,15]. In the trial of ND granules plus haloperidol compared with haloperidol only, both groups showed lassitude, drowsiness, and poor appetite. The adverse reaction rates were 13.3% in the ND granules plus haloperidol group and 36.7% in the haloperidol only group [17]. In the study of ND granules versus placebo, 6.1% of the participants reported loss of appetite and 3% experienced constipation in the ND granules group compared with no adverse reactions in the placebo group [16].

## Discussion

To our knowledge, this is the first systematic review of the effects of herbal medicines for tic disorders. The results suggested that QZR and ND granules were effective in treating tic disorders or Tourette’s syndrome. However, the evidence suggesting that QZR and ND granules represented an effective modality for treating tic disorders or Tourette’s syndrome was limited by small number of trials and the high risk of bias in primary trials.

Only two of the included trials reported randomisation methods, and only one used a placebo control. Concealment of treatment allocation was not reported in any of the studies. Trials with inadequate blinding and inadequate allocation concealment are likely to show exaggerated treatment effects [18], thus limiting the reliability of the study results.

In the four trials, the method for assessing the effects of QZR and ND granules was identical in using the YGTSS score. However, the YGTSS scores in the two Tourette’s syndrome studies were much lower than those in the two QZR studies. These observations indicated that Wu’s group selected severe tic disorder cases, although Tourette’s syndrome is generally more severe than tic disorders. Two trials seemed to be duplicate publications. They had the same methodology and outcome measures, except for the sample size, and the same first author, and were performed within similar periods of time. We contacted the authors to clarify these points, and the answer was that one paper described results from a multicentre study and the other reported results from a single-centre study. The authors might have included different numbers of sub-centres in the two articles. We also requested clarification for the other two studies with regard to whether they might possibly describe four-armed trials, but the authors did not answer. Even if this were true, it would not overrule our conclusion.

QZR and ND granules appeared to have potential for treating patients with tic disorders. There was also other evidence of their pharmaceutical effects and underlying mechanisms. QZR improved tic behaviour in mouse and rat models [10,19] while ND granules were effective for treating attention-deficit/hyperactivity disorder in an RCT and inhibited stereotypic behaviour in a Tourette’s syndrome rat model by modulating dopamine production and expression of the dopamine D2 receptor gene [9,20,21]. *Gastrodia elata* Blume is the main herb in both QZR and ND granules [14-17]. It had neuroprotective effects on human dopaminergic SH-SY5Y cells and an antidepressant-like effect *via* regulation of both the serotonergic and dopaminergic systems in a rat model [22,23].

Although our search strategy might locate all relevant data, there might be some unidentified studies and unpublished studies with negative results. Unpublished RCTs with negative results could provide different outcome with this review [24].

## Conclusions

This systematic review provided first piece of limited meta-analytic evidence for the effectiveness of herbal medicines in improving the symptoms of tic disorders.

## Competing interests

The authors declare that they have no competing interests.

## Authors’ contributions

YHK, MSL, CKS, HSL, and HWL conceived and designed the study. YHK, CKS, BCK, HWL, and MSL searched and selected the trials, and extracted, analysed, and interpreted the data. YHK, MSL, CKS, HSL, and HYL wrote the manuscript. All authors read and approved the final version of the manuscript.
